# Human Nasal Cells in Nanofibrillar Cellulose Hydrogel: Viability, Function, and Implications for Bone Tissue Regeneration

**DOI:** 10.3390/cells15070641

**Published:** 2026-04-02

**Authors:** Marijana Sekulic, Alina Korah, Simona Negoias, Daniel Bodmer, Vesna Petkovic

**Affiliations:** 1 Department of Biomedicine, University of Basel, University Hospital Basel, 4031 Basel, Switzerland; 2 Faculty of Medicine, University of Basel, 4056 Basel, Switzerland; 3 Otorhinolaringology Department, University Hospital Basel, 4031 Basel, Switzerland

**Keywords:** nasal epithelial cells, bone tissue regeneration, nanofibrillar cellulose hydrogel, TEER, permeability

## Abstract

Endoscopic sinus surgery (ESS) is commonly performed to treat chronic rhinosinusitis and selected sinonasal tumors, yet postoperative complications such as neo-osteogenesis and restenosis remain frequent, largely due to impaired mucosal regeneration after extensive epithelial and bony tissue loss. Successful nasal epithelial repair requires a microenvironment that preserves cell viability, phenotype, and barrier integrity. Conventional culture substrates often lack physiological relevance or rely on animal-derived components, limiting translational applicability. In this study, we evaluated nanofibrillar cellulose (NFC) hydrogel (GrowDex^®^) as a xeno-free scaffold for primary human nasal epithelial cells (NECs). NECs isolated from healthy donor tissue were characterized by immunofluorescence and qPCR for basal, goblet, and ciliated cell markers. Cells embedded in NFC were assessed for viability, cytotoxicity, epithelial morphology, and barrier function. Transepithelial electrical resistance (TEER) and FITC-dextran permeability assays were used to quantify barrier integrity and compared with collagen- and polylysine-based controls. NECs cultured in NFC maintained high viability, stable epithelial morphology, and preserved subtype-specific marker expression without detectable cytotoxicity. NFC-supported cultures demonstrated enhanced barrier formation, indicated by higher TEER values and reduced paracellular permeability relative to controls, and sustained structural integrity during extended culture. These findings identify NFC hydrogel as a biocompatible, non-animal scaffold that supports functional human nasal epithelium regeneration and may contribute to advanced tissue engineering strategies for craniofacial bone repair.

## 1. Introduction

Injury to the sinonasal mucosa, particularly when bone is exposed, creates a hostile tissue environment that impairs healing and predisposes to complications such as abnormal bone remodeling, impaired drainage, and secondary pathologies [1,2,3]. The introduction of endoscopic sinus surgery (ESS) has enabled minimally invasive access to sinonasal tissues and transformed disease management. However, extensive mucosal removal or bone exposure in advanced chronic rhinosinusitis and tumor cases increases the risk of aberrant wound healing, characterized by fibrosis, neo-osteogenesis, and stenosis [4]. Current strategies, such as mucosal flaps or grafts, provide only partial coverage and remain limited in their ability to restore a stable, functional mucosal barrier [5]. At the biological level, the injured sinonasal microenvironment is marked by persistent inflammation, oxidative stress, and hypoxia. These conditions compromise epithelial regeneration and reduce the survival of transplanted cells, limiting the effectiveness of cell-based therapies. Recent efforts therefore focus on biomaterial-based approaches that can recreate a protective, pro-regenerative niche. Optimized scaffolds not only provide structural support but also modulate local cues, improve cell viability, and promote integration, offering new avenues for enhancing tissue repair and long-term mucosal function [6].

Hydrogels are water-rich, 3D polymer networks that mimic natural tissue, making them ideal for biomedical use. Their structure supports drug delivery, wound healing, and tissue engineering by enabling controlled release and promoting cell growth [7]. Nanofibrillar cellulose (NFC) is an advanced form of cellulose that has at least one dimension in the nanometer range. What makes it particularly interesting is how it combines the well-known benefits of cellulose, biocompatibility, biodegradability, and renewability, with the unique properties of nanomaterials. NFC’s low density, high strength, and large surface area make it ideal for biomedical applications [8].

This study investigated the potential application of NFC hydrogel as a scaffold for cultured primary human nasal epithelial cells (NECs). The long-term aim is to develop an autologous transplant material for use in covering exposed bone and preventing postoperative bone overgrowth following radical sinonasal surgeries. NFC was selected for its consistent and well-defined composition, which is critical when considering biomaterials for clinical use. It is biologically inert (i.e., resistant to degradation and non-immunogenic in the human body) and exhibits excellent biocompatibility. In addition, NFC gel is stable at room temperature, making it suitable for storage and use in clinical environments, including operating rooms. Its viscosity can be reversibly decreased through gentle mechanical agitation, such as repeated movement of a syringe plunger, transitioning the material from gel-like to a more liquid state, facilitating handling and application [9,10].

Here, we used non-animal derived UPM NFC hydrogel GrowDex™ and cultured primary human nasal cells under different gel concentrations in order to follow nasal cell growth and properties over time to evaluate the suitability of this gel for human use. UPM’s NFC hydrogel is derived from birch wood and composed entirely of plant-based components and water, offering a sustainable and xeno-free alternative for biomedical applications. This hydrogel exhibits favorable viscoelastic characteristics, shear-thinning behavior, and rapid recovery after mechanical stress, making it suitable for injectable formats and 3D cell culture systems [9,10].

This study aimed to evaluate the potential of NFC wound dressing as a scaffold for human NECs with the goal of developing a cell transplantation approach that is entirely free of animal-derived components. Such a method could be used in wound treatment and to prevent bone overgrowth following radical tissue removal surgeries.

## 2. Materials and Methods

### 2.1. Isolation and Cultivation of Human Nasal Epithelial Cells

Patient nasal tissue was collected during surgery, placed in a sterile tube containing nasal epithelial medium (PromoCell, Heidelberg, Germany, cat#c-21060), and immediately used for further processing in the cell culture lab. Donors comprised both genders and ranged in age from 30 to 65 years (6 independent donors). Each cell culture was derived from a single donor and treated as an independent biological replicate, resulting in a total of *n* = 6 biological replicates across experiments. No pooling of cells between donors was performed, and cells were used between passages 2 and 3. Healthy sinonasal epithelial samples were obtained intraoperatively from patients undergoing routine endoscopic surgery. Specifically, tissue was collected from the sphenoid sinus mucosa in patients undergoing endoscopic transsphenoidal pituitary surgery, and from the medial aspect of the middle turbinate in patients undergoing concha bullosa resection. In all cases, biopsies were taken from macroscopically normal, non-inflamed mucosa under endoscopic guidance. No ethical permit was required for this type of anonymized sample collection and for the scope of our study (details are provided in the statement on ethics approval and consent). The tissue was cut into small (1–2 mm) pieces and exposed to 0.25% Trypsin-EDTA (Sigma, St. Louis, MO, USA) for 5 min at 37 °C. Upon incubation with trypsin, the reaction was stopped with a blocking solution containing 10% FBS. The suspension was centrifuged at 1500 rpm for 5 min, the supernatant removed, and cell tissue pieces resuspended in nasal epithelial medium and distributed in a collagen-coated 24-well plate. The plates were incubated at 37 °C in 5% CO_2_, changing the medium every 2 days. After approximately 1 week, at full confluency, the cells were transferred to a 25 cm^2^ collagen-coated flask (T-25 flask Thermo Fisher, Waltham, MA, USA) to allow further expansion. A sample of the cells was stained with pan-cytokeratin to confirm phenotype before further use or stored in liquid nitrogen.

### 2.2. Quantitative PCR

RNA was isolated from collected cells and extracted using the Direct-Zol RNA MiniPrep Kit (Zymo Research, Irvine, CA, USA; cat#R2050) according to the manufacturer’s instructions. Total RNA (1000 ng) was reverse-transcribed using a High-Capacity cDNA Reverse Transcription Kit (Applied Biosystems, Waltham, MA, USA). We analyzed triplicate samples by quantitative PCR on an ABI Prism 7900HT Sequence Detection System (Applied Biosystems) using the Power SYBR Green Master Mix (Applied Biosystems, Applied Biosystems, Waltham, MA, USA). Primers targeting GAPDH were synthesized by Microsynth (Balgach, Switzerland) and added at a final concentration of 250 nM per reaction. The full 5′–3′ primer sequences used in this study were: *KRT1* forward, AGAGTGGACCAACTGAAGAGT; *KRT1* reverse, ATTCTCTGCATTTGTCCGCTT; *KRT19* forward, ACCAAGTTTGAGACGGAACAG; *KRT19* reverse, CCCTCAGCGTACTGATTTCCT; *MUC5AC* forward, CAGCACAACCCCTGTTTCAAA; *MUC5AC* reverse, GCGCACAGAGGATGACAGT; *CLDN1* forward, CCTCCTGGGAGTGATAGCAAT; *CLDN1* reverse, GGCAACTAAAATAGCCAGACCT; *OCLN* forward, ACAAGCGGTTTTATCCAGAGTC; *OCLN* reverse, GTCATCCACAGGCGAAGTTAAT; *TJP1* forward, CAACATACAGTGACGCTTCACA; *TJP1* reverse, CACTATTGACGTTTCCCCACTC; *GAPDH* forward, GGAGCGAGATCCCTCCAAAAT; *GAPDH* reverse, GGCTGTTGTCATACTTCTCATGG. The relative quantities of specifically amplified cDNAs were calculated by the comparative threshold cycle method (2^−∆∆Ct^) using *GAPDH* expression as the endogenous reference.

### 2.3. Cytotoxicity Assay

The LDH-Glo™ Cytotoxicity Assay (Promega, Madison, WI, USA; cat. #J2381) was used to assess cytotoxicity by quantifying lactate dehydrogenase (LDH) released from damaged cells due to compromised cell membrane integrity. Samples were processed according to the manufacturer’s instructions. Briefly, 2.5 µL of cell treatment or control medium was mixed with 47.5 µL of LDH Storage Buffer in a 96-well plate (Corning Costar^®^, Corning, NY, USA; cat. #3917). Subsequently, 50 µL of LDH detection reagent mix was added to each well. The plate was incubated for 60 min at room temperature, after which luminescence was measured using a plate reader (BioTek Synergy H1, Winooski, VT, USA).

### 2.4. TEER

The transepithelial electrical resistance (TEER) was measured with a Voltohmmeter EVOM3 (World Precision Instruments, Sarasota, FL, USA). After cells were counted by Trypan Blue staining in an automated cell counter (Bio-Rad, Hercules, CA, USA, TC20), the cell suspension was placed on a Transwell^®^ (Corning, Corning, NY, USA) membrane. The NEC suspension was added on the luminal side of the insert and left for 3 h to attach in the small suspension volume of 150 μL before the rest of the NEC medium was added.

For TEER measurements, NECs were seeded at a density of 2 × 10^5^/cm^2^ and grown on membrane inserts (Corning, Corning, NY, USA; cat# 3470) coated with either NFC hydrogel or polylysine. Resistance measurements were performed in accordance with the manufacturer’s guidelines (WPI, EVOM3). To ensure consistent electrode performance, probe tips were cleaned weekly by immersion in 1% Tergazyme^®^ (Alconox, White Plains, NY, USA) for 15 min, followed by thorough rinsing with sterile water. The same cleaning procedure was carried out immediately prior to disinfection and at the start of each experimental run. For sterilization, STX4 electrodes were exposed to 70% ethanol for up to 5 min and subsequently washed with either culture medium or phosphate-buffered saline (PBS). Electrical resistance was then recorded for both experimental and control conditions. Cells were seeded and allowed to adhere for 24 h before initial measurements were taken. Prior to use, electrodes were equilibrated in culture medium for several minutes. Background resistance was determined using cell-free Transwell^®^ inserts containing only epithelial cell medium. Upon completion of measurements, electrodes were again disinfected with ethanol, rinsed with sterile water, and left to air-dry. TEER values were calculated by subtracting the blank resistance (cell-free insert) from the recorded values and multiplying the resulting net resistance by the membrane surface area (cm^2^) of the Transwell^®^ inserts.

### 2.5. Permeability Assay

Nasal epithelial cells (NECs) were seeded onto 24-well Transwell^®^ inserts as previously described. Following 8 days of cultivation, once transepithelial electrical resistance (TEER) had stabilized, permeability assessment was performed using 70 kDa FITC-labeled dextran applied to the apical chamber. After a 2 h incubation period, samples were collected from the basolateral compartment, and fluorescence was quantified using a plate reader (BioTek Synergy H1) at excitation and emission wavelengths of 490 nm and 520 nm, respectively. Dextran concentrations were determined based on a calibration curve generated from known standards.

### 2.6. ICC Fluorescent Staining

NECs were cultured on 4-well glass-bottom plates (Ibidi, Fitchburg, WI, USA; cat. #80426). Cells were fixed using 4% paraformaldehyde (Sigma-Aldrich, Burlington, MA, USA; cat. #158127) prepared in PBS (Sigma; cat. #P4417), followed by permeabilization with 0.1% Triton X-100 (Sigma; cat. #X100). Samples were then incubated for 1 h at room temperature with primary antibodies targeting MUC5AC (Invitrogen; Carlsbad, CA, USA, cat. #MA5-12178), pan-cytokeratin (Invitrogen; cat. #CK102), FOXJ1 (Invitrogen; cat. #14-9965-82), and TP63 (Invitrogen; cat. #703809). After washing, appropriate secondary antibodies conjugated to Alexa Fluor 488 (Invitrogen; cat. #704060 and A-11001) were applied for 1 h at room temperature together with rhodamine-phalloidin (Invitrogen; cat. #R415). Following additional PBS washes, nuclei were stained with DAPI for 5 min. Samples were then rinsed and imaged using a Nikon Eclipse Ti2 inverted widefield microscope (Nikon, Tokyo, Japan). Image processing and analysis were carried out with Fiji (Win32, version 2.0.0-rc-49/1.51d).

### 2.7. Live-Cell Staining

To further monitor cell viability, live-cell staining was performed using CellTracker™ CMFDA dye (Thermo Fisher Scientific; Waltham, MA, USA, cat#C2925). NECs were cultured in 48-well plates coated with either NFC hydrogel or collagen. Live staining was carried out on days 1, 7, 15, 21, and 28 according to the manufacturer’s instructions to assess cell viability and distribution over time.

### 2.8. Statistical Analysis

Statistical evaluations were conducted using GraphPad Prism (version 10.0.3 (217), San Diego, CA, USA). Comparisons involving more than two groups were analyzed using one-way or two-way ANOVA, applying Dunn’s multiple comparisons test and the Geisser–Greenhouse correction where appropriate. For pairwise comparisons, the Mann–Whitney test was employed with Welch’s correction. Normality of the data distribution was assessed using the Shapiro–Wilk test. A *p*-value < 0.05 was considered statistically significant.

## 3. Results

### 3.1. Validation of Nasal Epithelial Cell Phenotype

**Figure 1:**
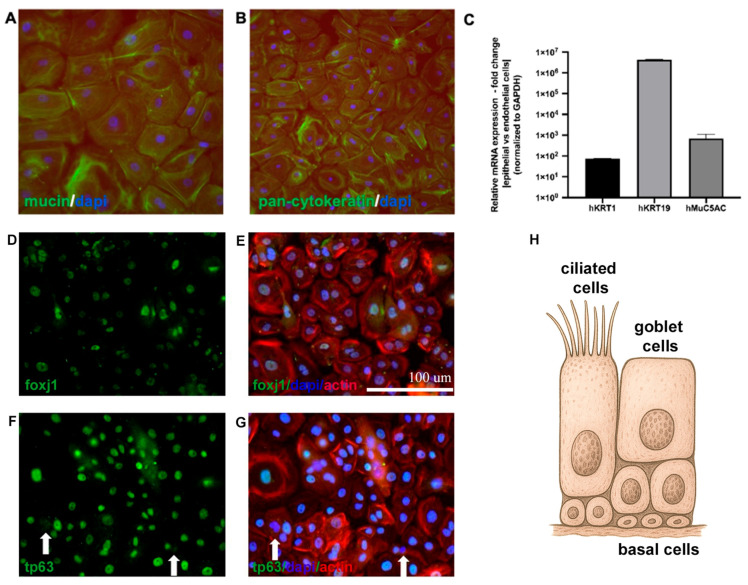
Phenotypic characterization of human nasal epithelial cells by gene expression and immunofluorescence. (**A**,**B**) Representative immunofluorescence images of cultured primary human nasal epithelial cells. Robust expression of epithelial-specific protein markers MUC5AC and pan-cytokeratin confirms epithelial identity. (**C**) Quantitative gene expression analysis showing increased expression of epithelial marker genes (*MUC5AC*, *KRT1*, *KRT19*) in nasal epithelial cells compared to human endothelial cells, supporting cell-type specificity at the transcriptional level (*n* = 3). (**D**,**E**) Immunofluorescent staining of FOXJ1 (green), a marker of ciliated cells, shown alone and merged with DAPI (nuclei, blue) and phalloidin (actin, red) staining. (**F**,**G**) Immunofluorescent staining of TP63 (green), a marker of basal cells, shown alone and merged with DAPI (blue) and phalloidin (actin, red). White arrows indicate TP63-negative cells within the culture. (**H**) Schematic representation of the three main nasal epithelial cell subtypes identified in the donor-derived primary nasal epithelial cell cultures (created using ChatGPT, version 5.4).

To evaluate whether the nasal epithelial phenotype was preserved following the isolation of cells from donor nasal tissue, we assessed both the protein and gene expression levels. Immunofluorescent staining with epithelial-specific antibodies against MUC5AC and pan-cytokeratin confirmed that cultured NECs maintained expression of mucin and cytokeratin proteins characteristic of the respiratory epithelium. These markers were detected in cells isolated from both the nasal septum and turbinate regions, indicating preservation of nasal epithelial identity across anatomical sites. To assess the presence of specific nasal epithelial subtypes, additional immunofluorescent staining was performed for FOXJ1, a marker of ciliated cells, and TP63, a marker of basal cells. Positive staining for both markers confirmed the presence of ciliated and basal cell populations within the cultures (Figure 1). To validate these findings at the transcriptome level, quantitative PCR analysis demonstrated robust expression of epithelial-specific genes (*KRT1*, *KRT19*, and *MUC5AC*), further supporting maintenance of the epithelial phenotype in vitro (Figure 1C).

### 3.2. NFC-Based Hydrogel Suitability for Nasal Epithelial Cell Growth

**Figure 2:**
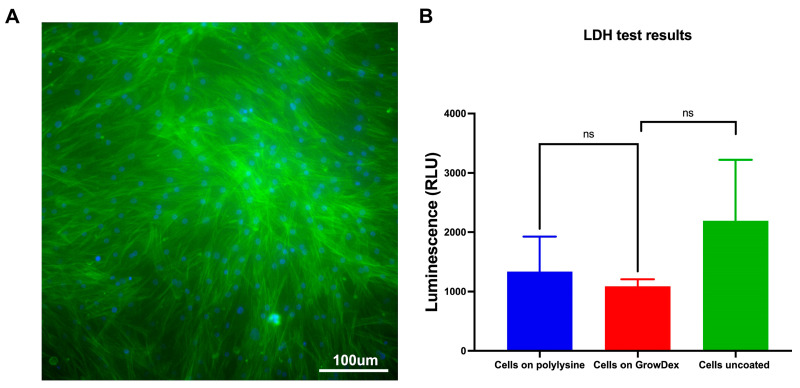
Nanofibrillar cellulose hydrogel (GrowDex) supports cell viability and shows no signs of cytotoxicity in nasal epithelial cells (NECs). (**A**) Live-cell imaging using CellTracker™ CMFDA dye confirmed the high viability and intact cytoplasmic morphology of NECs cultured in GrowDex hydrogel. (**B**) Lactate dehydrogenase (LDH) cytotoxicity assay shows no significant differences in LDH release between NECs cultured on uncoated surfaces, poly-L-lysine-coated surfaces, or in GrowDex, indicating the absence of cytotoxic effects. Data are presented as mean ± SD (*n* = 6); ns, not significant.

To evaluate the suitability of the NFC-based GrowDex^®^ hydrogel for supporting nasal epithelial cell culture, we assessed the viability and morphology of isolated human NECs embedded within the hydrogel. Live-cell staining with CellTracker™ CMFDA dye was performed 72 h after seeding. As shown in Figure 2A, NECs exhibited strong viability and well-preserved cytoplasmic integrity, indicating that GrowDex provides a biocompatible and supportive microenvironment for nasal epithelial cell survival and maintenance over time.

In addition, no significant differences in LDH release were observed between NECs cultured on uncoated plastic, cultured on poly-L-lysine-coated surfaces, or embedded within GrowDex, confirming the non-toxic nature of the hydrogel (Figure 2B).

### 3.3. Nasal Epithelial Barrier Formation and Permeability in Nanofibrillar Cellulose Hydrogel

**Figure 3:**
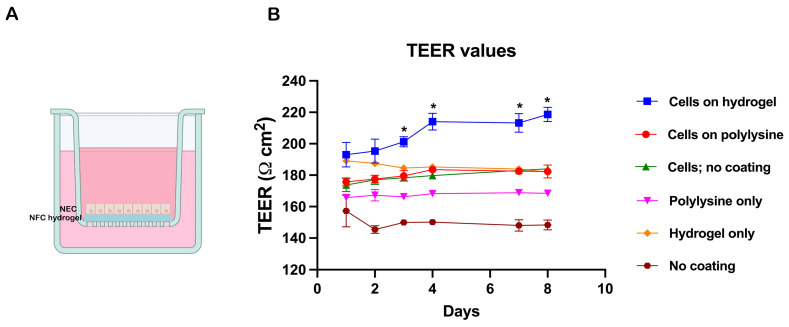
Measurement of nasal epithelial barrier formation in nanofibrillar cellulose (NFC) hydrogel in Transwell culture using TEER. (**A**) Schematic representation of the Transwell setup showing nasal epithelial cells (NECs) embedded in GrowDex hydrogel on a 0.4 μm porous membrane. (**B**) Transepithelial electrical resistance (TEER) values measured over 8 days across different culture conditions. NECs grown in GrowDex hydrogel exhibited significantly higher TEER compared to cells grown on poly-L-lysine-coated or uncoated membranes, indicating enhanced epithelial barrier integrity. Data are presented as mean ± SD (*n* = 6); * *p* < 0.05.

To evaluate the ability of NECs to form a functional epithelial barrier within the GrowDex hydrogel, cells were seeded into Transwell inserts with 0.4 μm porous membranes (Figure 3A). Barrier integrity was assessed using TEER, a non-destructive, real-time method for monitoring epithelial tight junction formation. TEER values were recorded daily over an 8-day period.

In addition to the GrowDex condition, NECs were cultured on poly-L-lysine-coated and uncoated Transwell membranes. Control inserts without cells, either uncoated or coated with poly-L-lysine or GrowDex, were also included to account for background conductivity. Among all tested conditions, NECs cultured in GrowDex exhibited the highest TEER values, indicating superior epithelial barrier formation and integrity compared to cells grown on coated or uncoated membranes (Figure 3B).

### 3.4. Nanofibrillar Cellulose Hydrogel Supports Superior Nasal Epithelial Barrier Function and Long-Term Culture Viability

**Figure 4:**
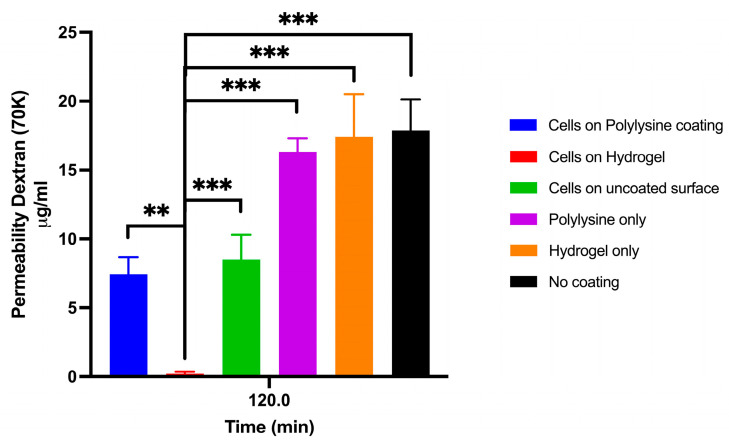
Nanofibrillar cellulose (NFC) hydrogel supports superior nasal epithelial barrier integrity and reduced permeability. Cells cultured on NFC hydrogel exhibited significantly enhanced barrier function, demonstrating lower permeability to FITC-dextran compared to all other tested conditions, including cells grown on poly-L-lysine coating, uncoated membranes, and no coating. Data are presented as mean ± SD (*n* = 6); ** *p* < 0.01, *** *p* < 0.001.

NECs seeded onto Transwell membranes coated with NFC hydrogel, coated with poly-L-lysine, or left uncoated (treated for optimal cell attachment) were assessed for epithelial barrier function based on 70 KDa FITC-dextran permeability after 8 days in culture. NECs cultured with NFC demonstrated significantly higher barrier integrity, which was reflected in the lowest permeability compared to all other conditions, including NECs grown on poly-L-lysine, hydrogel only, poly-L-lysine only, and uncoated Transwells (Figure 4).

**Figure 5:**
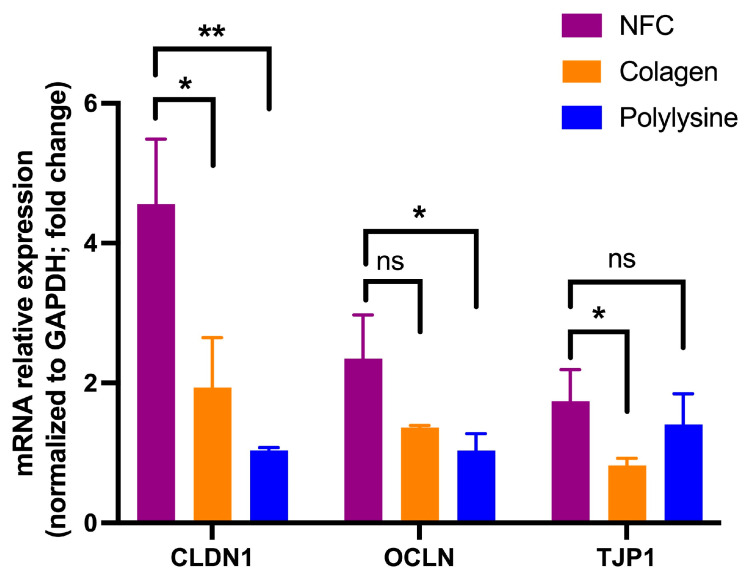
Expression of tight junction genes in nasal epithelial cells (NECs) cultured on different substrates. NECs were cultured for 72 h on nanofibrillar cellulose (NFC), collagen, or poly-L-lysine. mRNA expression levels of *CLDN1*, *OCLN*, and *TJP1* were quantified by qPCR and normalized to *GAPDH* (fold change). Data are presented as mean ± SD (*n* = 3); * *p* < 0.05, ** *p* < 0.01, ns, not significant.

Expression of tight junction genes was modulated by the substrate on which nasal epithelial cells were cultured. *CLDN1* expression was significantly increased in cells grown on NFC compared to both collagen (*p* < 0.05) and poly-L-lysine (*p* < 0.01). For *OCLN*, a moderate increase was observed in NFC compared to poly-L-lysine (*p* < 0.05), while no significant difference was detected between NFC and collagen. In contrast, *TJP1* expression was significantly higher in NFC compared to collagen (*p* < 0.05), whereas no significant difference was observed between NFC and poly-L-lysine. Overall, NFC supported equal or higher expression of key tight junction genes compared to conventional culture substrates (Figure 5).

**Figure 6:**
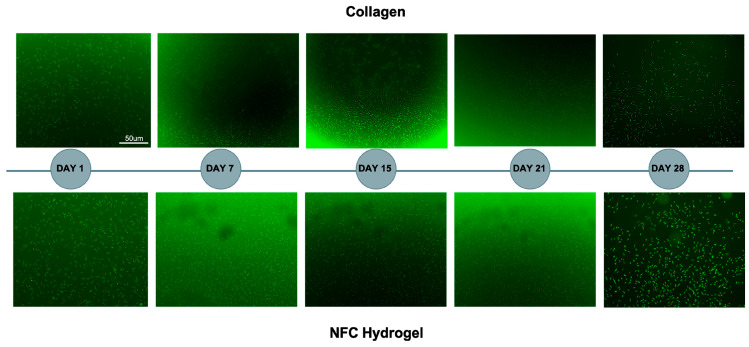
Long-term growth and viability of nasal epithelial cells (NECs) embedded in nanofibrillar cellulose (NFC) hydrogel (GrowDex™). NECs were stained with CellTracker™ CMFDA live-cell dye on days 1, 7, 15, 24, and 28 to monitor cell viability and distribution within the NFC hydrogel over time. For comparison, NECs cultured in a collagen matrix were included as a reference condition.

To assess NEC viability and spatial distribution during extended culture, cells embedded in NFC hydrogel were stained with CellTracker™ CMFDA live-cell dye on days 1, 7, 15, 24, and 28. NECs cultured within the NFC hydrogel maintained robust viability and uniform distribution throughout the 28-day period, comparable to NECs grown on a collagen matrix under the same conditions (Figure 6).

## 4. Discussion

Endoscopic sinus surgery is the standard technique, but in refractory cases more extensive procedures are performed, often requiring broad mucosal resection and bone exposure, which substantially increase the risk of complications such as abnormal bone growth, stenosis, and mucocele formation [11,12,13]. Maintaining a moist environment is essential for optimal wound healing, and hydrogel-based wound dressings have proven effective in supporting this process [14]. Conventional hydrogels suffer from several intrinsic limitations, including low mechanical strength, poor processability, inhomogeneous network formation, and minimal energy dissipation, which restrict their utility across a broad spectrum of applications [15,16]. These shortcomings arise from their loosely cross-linked, single-network architectures, which are prone to structural heterogeneity and brittleness [17]. Matrigel^®^ is a widely used cell culture matrix derived from EHS mouse sarcoma cells, rich in ECM proteins and growth factors. Its thermal properties allow it to remain liquid at 4 °C and solidify around 10 °C, making it useful for cell transport at room temperature [18]. A major limitation of this hydrogel lies in its animal-derived components and undefined composition, which, combined with the need for storage and handling at 4 °C, render it unsuitable for applications in human tissue repair [19]. Among various hydrogel materials, nanofibrillar cellulose (NFC) stands out due to its excellent biocompatibility, resistance to enzymatic degradation, non-immunogenicity in the human body, and the absence of animal-derived components. It is a well-defined, two-component system that is modifiable, structurally stable, and does not require temperature-dependent handling, making it particularly suited for biomedical applications.

This study explored autologous NECs in a non-animal NFC hydrogel for potential nasal mucosal repair. NECs were isolated and characterized, showing preserved nasal epithelial subtypes, including goblet (MUC5AC+), ciliated (FOXJ1+), and basal (TP63+) cells, with strong pan-cytokeratin expression confirming epithelial identity. Subsequently, we combined the NFC hydrogel with isolated NECs and assessed cell viability and potential cytotoxicity within the gel. Following isolation from donor tissue and confirmation of the nasal epithelial phenotype, the embedded cells demonstrated excellent viability and showed no signs of cytotoxicity. These findings are consistent with previous reports indicating good cell compatibility of NFC hydrogels for various cell types, including adipose-derived cells, and now extend this evidence to human NECs [20,21].

We also followed functional aspects of nasal epithelial barrier formation as a fundamental characteristic of nasal epithelial cells in the NFC hydrogel by measuring the TEER and permeability to 70 KDa FITC-dextran, which showed superiority compared to a standard coating, such as collagen or poly-L-lysine. The observed upregulation of tight junction genes (*CLDN1*, *OCLN*, and *TJP1*) in NECs cultured on NFC suggests that this biomaterial provides a microenvironment conducive to epithelial barrier formation. In particular, the robust increase in *CLDN1* expression indicates enhanced potential for tight junction assembly and paracellular sealing. While *OCLN* and *TJP1* showed more moderate or substrate-dependent differences, their overall expression levels in NFC were comparable to or higher than standard coatings, supporting the ability of NFC to maintain epithelial integrity. These findings align with the hypothesis that nanocellulose-based scaffolds can promote physiologically relevant epithelial phenotypes, potentially through favorable surface topography and cell–matrix interactions. Importantly, the absence of reduced tight junction gene expression further supports the biocompatibility of NFC.

Studies culturing human NECs on collagen matrix supports have found that, though a good barrier forms, the tight junction integrity could vary depending on culture conditions, implying that alternative matrices could yield improved consistency [22]. An analysis of different culture conditions with the NEC line RPMI 2650 showed that the culture interface and coating strongly influenced TEER and FITC-dextran permeability, indicating that advanced supports or hydrogels may better mimic native tight barriers [23]. Another study confirmed that the air–liquid interface (ALI) and appropriate matrix coatings significantly impact TEER and permeability in human NECs, supporting the need for innovative hydrogels to enhance barrier formation [24]. ALI collagen-based systems are associated with limitations such as animal-derived origin, batch-to-batch variability, and limited tunability of mechanical and structural properties. In contrast, the NFC hydrogel used in this study provides a xeno-free, highly reproducible, and structurally tunable scaffold. Importantly, our results demonstrate that NECs cultured within NFC maintain viability, epithelial phenotype, and barrier-associated functional properties, including tight junction formation and controlled macromolecular permeability. Taken together, while ALI collagen-based systems remain the benchmark for in vitro nasal epithelial differentiation, our findings suggest that NFC hydrogels represent a promising alternative platform, particularly in the context of tissue engineering and regenerative applications, where reproducibility, biocompatibility, and clinical translatability are critical. Moreover, the ability of NFC to support functional barrier formation, as assessed by both TEER and permeability assays, underscores the importance of combining complementary readouts to fully characterize epithelial integrity and transport function.

Long-term survival of the NECs was one of the concerns we addressed. To this end, we followed cell viability in the NFC hydrogel over a period of several weeks, finding that cell viability was unaffected with great growth in the NFC hydrogel environment (Figure 6). These findings are similar to other studies that found that NFC hydrogel provides a supportive 3D scaffold for long-term culture of human adipose-derived mesenchymal stromal cells, maintaining high cell viability and angiogenic potential over extended periods [20]. Similarly, NFC hydrogel was shown to promote the 3D culture of liver cells, supporting long-term survival, polarization, and function without added growth factors, highlighting its excellent biocompatibility as an animal-free scaffold [25].

One potential concern regarding the use of NFC hydrogels is the human body’s inability to naturally degrade cellulose, which could pose challenges if removal or remodeling of the material is required. In such cases, the enzyme cellulase (e.g., GrowDase™) can be employed, as it effectively degrades NFC into glucose without harming embedded or surrounding cells. While cellulase can be used experimentally to degrade NFC, its clinical applicability in the sinonasal cavity has not yet been established and requires further evaluation. Previous studies provide indirect evidence supporting the biocompatibility of cellulase-mediated scaffold degradation. Continuous sheets of endothelial cells, fibroblasts, and functionally competent cardiac cell networks have been successfully cultured on cellulose-based scaffolds and subsequently released via cellulase treatment without compromising cell integrity or function. Studies of wound healing have demonstrated that cellulase-assisted degradation of NFC hydrogels enables the controlled release of bioactive compounds while preserving the viability of adjacent skin cells, underscoring the enzyme’s biocompatibility and cell-friendly nature [26,27,28,29]. These findings suggest that cellulase can be applied under controlled conditions without inducing overt cellular damage, although its safety in the sinonasal environment remains to be specifically evaluated.

## 5. Conclusions

The results of our study demonstrate that NECs derived from human nasal mucosa and embedded in nanofibrillar cellulose hydrogel form a supportive, xeno-free construct that maintains cell viability, preserves basal, goblet, and ciliated subtypes, and promotes robust barrier formation. Collectively, these findings establish the NFC–NEC system as a reproducible and physiologically relevant in vitro model of human nasal epithelium with preserved cellular heterogeneity and barrier integrity. The defined composition, structural stability, and enzymatic degradability of the NFC hydrogel further support its suitability as a controlled microenvironment for epithelial culture and investigation of regeneration-related processes, where barrier restoration is critical for optimal bone repair.

While the NFC–NEC system demonstrates promising characteristics for epithelial regeneration, it is important to note that the present findings are based on in vitro observations. From a translational perspective, this platform may provide a foundation for future tissue-engineering strategies, including applications in sinonasal and broader craniofacial reconstruction, where coordinated regeneration of epithelial and underlying tissues is required following tumor resection or traumatic injury. Future studies incorporating more complex experimental settings, including co-culture models with osteogenic components and in vivo validation, will be necessary to further assess its role in post-surgical tissue repair and remodeling.

## Data Availability

The raw data supporting the conclusions of this article will be made available by the authors upon request.
